# 

**Published:** 1893-07-08

**Authors:** 


### CONTENTS.

**The Present Position of Nursing** . . . . . . 225

**Medical History from the Earliest Times.** By E. T. Withington, M.A., M.B.Oxon. LXI.—The Organicists . . 227

**Contemporary Theory and Research** . . . . . 228

**Our Weaker Brethren** . . . . . . . . . 229

**Gleanings in Science and Medicine** . . . . . . 230

**The Hospital Clinic—**

Glasgow Western Infirmary.—Arrangement for Electrical Treatment . . . . . . . . . . . 233

Some Simple Uses of Carbolic Acid. II. . . . . . . 234

Royal Hospital for Diseases of the Chest, City Road.—Treatment of Pulmonary Phthisis . . . . . . . 235

**Editor's Letter Box.**—Antiseptic Inunction in Scarlet Fever . 236

**Annotations.**—The "Drink-Crave Cure"—A Unique City Company—Summer Cobwebs and the Broom . . . . . 231

**Notes and News** . . . . . . . . . . 232

**The Institutional Workshop—**

Within the Institutions.—The Illinois Training School for Nurses. By Our Special Commissioner . . . . . . 237

Practical Departments:—Hygienic Teapots—Toilet Tray . 238

Festival Dinners and Meetings.—Earlswood Asylum—St. Mary's Hospital . . . . . . . . . 239

Construction Notes . . . . . . . . . 239

**Scraps and Gleanings** . . . . . . . . . 240

**The Book World of Medicine and Science** . . . vii

**Medical Vacancies and Appointments** . . . . . x

### EXTRA SUPPLEMENT.—THE NURSING MIRROR.

**News from the Nursing World.**—The Value of a Signature—Provident and Improvident—Sickroom Cookery—Practising and Preaching—District Nursing—Provisions for Convalescence—A Lost Opportunity—Qualified Medical Women—Short Items—The Disabled Nurse—"The Hospital" Endowed Bed . . . . . . . . . . cxli

**Sanitation for Nurses.** By A Sanitary Inspector. XII.—Disinfection and Disinfectants . . . . . . . cxliii

**The Royal Charter—Appointments—Where to Go** . cxliv

**Presentations** . . . . . . . . . . cxlvi

**The International Nursing Congress** . . . . . cxlv

**Everybody's Opinion.**—A Holiday Home Wanted—Small-pox—Robben Island: A Fortnight Amongst the Lepers—"Plain and Unattractive Duties" . . . . . . . . cxlvii

**Queen Victoria's Jubilee Institute for Nurses** . cxlvii

**Minor Appointments** . . . . . . . . . cxlvii

**Nursing in Workhouse Infirmaries.**—A Nurse's Qualifications . . . . . . . . . . . cxlviii

**Notes and Queries** . . . . . . . . . cxlviii

**The Book World for Women and Nurses** . . . cxlix

